# Case Report: Dramatic Cholestasis Responsive to Steroids in a Newborn Homozygous for H63D HFE Variant

**DOI:** 10.3389/fped.2022.930775

**Published:** 2022-07-08

**Authors:** Luca Filippi, Sara Tamagnini, Francesca Lorenzoni, Anna Caciotti, Amelia Morrone, Rosa Scaramuzzo

**Affiliations:** ^1^Department of Clinical and Experimental Medicine, University of Pisa, Pisa, Italy; ^2^Neonatology Unit, Azienda Ospedaliero-Universitaria Pisana, Pisa, Italy; ^3^Laboratory of Molecular Biology of Neurometabolic Diseases, Department of Neuroscience, Meyer Children’s Hospital, Florence, Italy; ^4^Department of NEUROFARBA, University of Florence, Florence, Italy

**Keywords:** newborn, hereditary hemochromatosis, liver failure, cholestasis, steroids

## Abstract

In a newborn with very precocious liver failure, cholestatic jaundice, and low γ-glutamyl transpeptidase, progressive hepatosplenomegaly induced a progressively worsening respiratory distress, that was successfully treated with steroids. Laboratory and genetic tests did not find any disease usually associated with neonatal cholestasis. However, the patient was positive for a homozygous mutation of the *HFE* gene, which is associated with hereditary hemochromatosis, a disease with typical onset in adulthood. Although no firm conclusions can be drawn from a single clinical case, this experience suggests that hereditary hemochromatosis could have played a role in the induction of this serious cholestasis, probably already arisen in the uterus. We suggest that hereditary hemochromatosis ought to be included in the panel of the possible causes of neonatal cholestasis and that steroids ought to be added to the pharmacological armamentarium for treating specific conditions which cause cholestasis in newborns.

## Introduction

Jaundice is a common clinical finding in newborns and is usually the result of the accumulation of unconjugated bilirubin. Neonatal cholestatic jaundice, however, is less common (1). Cholestasis affects approximately 1 in every 2,500 infants and is due to multitude of causes. In preterm newborns and in infants with short bowel syndrome or intestinal failure, liver damage is usually caused by prolonged parenteral nutrition. Other causes of conjugated bilirubin accumulation include anatomic abnormalities of the biliary system, in *primis* biliary atresia, genetic disorders, metabolic diseases, and α1-antitrypsin deficiency. Less frequently, cholestasis can occur because of infections, endocrine disorders, toxin and drug exposures, vascular malformations, or neoplasms (2).

We report a newborn with an exceptionally precocious and rapidly worsening cholestatic jaundice with low γ-glutamyl transpeptidase (γ-GT), which dramatically regressed with steroid treatment.

## Case Description

The patient was born by cesarean section for podalic presentation at 37 + 3 weeks of gestation and had unrelated Moroccan parents. The birth weight was 3,442 g and the Apgar score was 8–9 at 1 and 5 min. The pregnancy (the mother’s fifth) was otherwise normal; one miscarriage in the maternal history. The mother took vitaminic supplements containing 30 mg of iron every day throughout pregnancy.

At birth, petechial rash and hepatosplenomegaly were evident. Laboratory exams at 3 h of life showed hypoglycemia; hemoglobin, 19.2 g/dL; red blood count, 5.29 × 106/μL; reticulocytes, 409 × 106/μL (7.7%); thrombocytopenia (31,000/μL); bleeding diathesis with international normalized ratio (INR) of 2.93; fibrinogen, 91 mg/dL; increased aminotransferases (AST 626 U/L, ALT 291 U/L) (reference range < 50), with a precocious cholestatic jaundice (total bilirubin of 8.1 mg/dL, conjugated bilirubin of 8.0 mg/dL); normal γ-GT (22 U/L); and ferritin, 1,600 μg/L. A herpes infection or gestational alloimmune liver disease (GALD) was suspected, and acyclovir and immunoglobulin treatments were started, respectively, together with plasma infusion and glucose administration at 7 mg/kg/min.

Hepatitis B virus, HCV, HSV1-2, HIV, CMV, enterovirus, and parechovirus together with serologic IgM tests for EBV, toxoplasma, rubella, adenovirus, parvovirus B19, coxsackievirus were negative.

Ammonia, lactate, uric acid, alkaline phosphatase, amylase, lipase, creatine phosphokinase, and α1-antitrypsin were within the normal range. Alpha-fetoprotein was 6,461 μg/L. Galactosemia was excluded because cholestasis was begun before oral nutrition. Acylcarnitine and urinary organic acids were normal. The plasma amino acid profile was compatible with liver failure (increased levels of threonine, glutamine, glycine, phenylalanine, ornithine, and lysine). Biliary acids were 59.4 μmol/L (reference range < 10).

Throughout the first days of life, aminotransferases were high (AST 400–500 U/L, ALT 150–250 U/L), while cholestasis worsened despite ursodeoxycholic acid treatment, with γ-GT constantly low. Plasma infusions were stopped on the fourth day, with a satisfactory liver function (INR ≤ 1.5). Stools were normally colored. The perforin expression in cytotoxic lymphocytes was normal, as were the quantitative urinary mucopolysaccharides (measured although the clinical presentation without hydrops fetalis was not typical of mucopolysaccharidoses). Disorders of cholesterol biosynthesis were excluded by analysis of sterol profiles in blood and urine. Severe infantile acid sphingomyelinase (ASM) deficiency (commonly known as Niemann-Pick type A/B) and Gaucher syndromes were excluded, respectively, as the levels of chitotriosidase and the enzymatic activity of sphingomyelinase and β-glucocerebrosidase were normal. Considering the difficult laboratory diagnosis of congenital Niemann-Pick type C, a disease with typical neonatal cholestatic liver failure, a specific genetic evaluation of the causal genes was planned. Early onset lysosomal acid lipase (LAL) deficiency (Wolman disease) was excluded as the LAL activity was normal. A genome-wide screening using array-CGH did not reveal microdeletions or/microduplications. The peripheral blood smear was normal. The hormonal profile (TSH, free-T4, 17-OH progesterone, testosterone, androstenedione, DHEA, cortisol, ACTH, Insulin-like Growth Factor I) was within normal range. Neonatal lupus was excluded by analysis of maternal and neonatal immunologic profiles (the absence of antibodies to Ro/SSA, La/SSB, U1-nRNP, Sm, Scl-70, Jo-1, CENP-B, PCNA, AMA-M2, PM-Scl, DFS 70, nucleosomes, histons, RNP, antiphospholipid, anticardiolipin, and antinuclear antibodies).

During the first week of life, conjugated bilirubin progressively increased reaching 33 mg/dL, biliary acids increased to 154 μmol/L, and ferritin remained around 700–800 μg/L. An abdomen sonography demonstrated spleen enlargement (maximum length of 6 cm) and hepatomegaly until the iliac fossa, with diffuse hyperechogenic aspect and a visible gallbladder. The neurologic evaluation showed mild axial hypotonia, and the bottle feeding worsened. Hepatosplenomegaly induced respiratory distress and acidosis (arterial CO_2_ values increased to 90–95 mmHg), hindering spontaneous breathing and necessitating non-invasive respiratory support.

**FIGURE 1:**
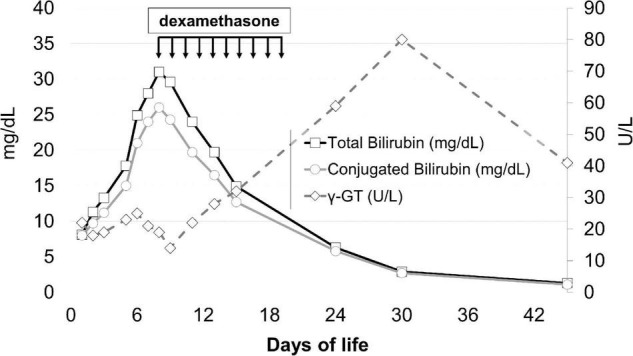
Total bilirubin, conjugated bilirubin, and γ-GT levels before and after steroid treatment. The arrows indicate the timing of steroid administration.

On day 9, upon the unlikely but not impossible hypothesis that such symptoms were caused by the neonatal onset of type 2 progressive familial intrahepatic cholestasis (PFIC2), which occasionally responds to steroids, dexamethasone of 0.3 mg/kg/12 h was started, with a dramatic clinical and laboratory response: general conditions rapidly improved, dyspnea and hypercapnia reverted within 24 h, and hepatosplenomegaly regressed in a few days. The values of conjugated bilirubin, bile acids, plasma ferritin, thrombocytopenia, and the coagulation profile improved, as did the levels of transaminases, although more slowly. Steroids were stopped after 10 days (Figure 1).

However, contrary to our hypothesis, genetic investigations did not confirm a diagnosis of PFIC, since no mutations in the ATP8B1, ABCB11, ABCB4, TJP2, and NR1H4 genes were found. Disorders potentially associated with cholestasis (Alagille syndrome, hereditary fructose intolerance, galactose disorders, Wilson disease, Niemann-Pick disease type C, Wolman disease, α1-AT, bile acid synthetic defects, mitochondrial DNA depletion syndrome, and peroxisome biogenesis disorders) were excluded by a genetic evaluation. Surprisingly, a homozygous mutation in the HFE gene (c.187C > G:p.His63Asp missense variants) was found, a genetic defect associated with hereditary hemochromatosis (HH), an autosomal recessive disease with typical onset in the fourth/fifth decade of life.

The infant was discharged at 2 months of life in good general health. He was bottle fed, and laboratory exams were normal, even though mild axial hypotonia persisted. The neurodevelopmental evaluation showed a poor motor repertoire, and the paroxysmal activity localized in the left lateral boundaries of the parietal-temporal-occipital regions was revealed by electroencephalography (EEG). A cerebral MR showed multiple areas of white matter damage in the left hemisphere, associated with a modest enlargement of the ventricular-cisternal system. Clinical follow-up at 5 months of life demonstrated an excellent hepatic function and a moderately reduced motor competence.

## Discussion

Neonatal cholestasis is a rare condition slowly progressive, but the extreme precocity and severity of cholestatic jaundice in this patient suggested a prenatal onset. The clinical signs associated with early liver failure, at first suggested a diagnosis of GALD, the most frequent manifestation of neonatal hemochromatosis due to a multi-organ iron accumulation, usually a consequence of an alloimmune maternal reaction against a fetal liver antigen (3). For this reason, treatment with intravenous immunoglobulins was immediately initiated. However, the precocious hepatosplenomegaly without signs of cirrhosis or portal hypertension, the high level of aminotransferases, the low values of α-fetoprotein, together with the progressive bilirubin overload and hepatosplenomegaly despite treatment did not support this hypothesis. The exclusion of the most frequent causes of neonatal cholestasis associated with low γ-GT prompted us to also consider typical diseases of adulthood, such as PFIC.

PFIC1 and PFIC2 are both characterized by low-serum γ-GT, although clinical manifestations usually begin in infancy and progress to biliary cirrhosis in the first/second decade of life (4). PFIC1 patients carry mutations in the ATP8B1 gene, whose protein stabilizes the integrity of the canalicular membrane (5). Patients with PFIC2 have a mutated bile salt export pump (Bsep) gene (i.e., the ABCB11 gene), the pivotal transporter of bile salt into the bile canaliculi (6). Studies performed in rat hepatocytes demonstrated that dexamethasone upregulates the mRNA levels of Bsep (7), an effect confirmed in rat livers (8). These data supported the successful use of steroids in two young patients carrying mutations in the gene encoding Bsep (9).

Because of the rapid clinical deterioration of our patient and following the hypothesis, albeit unlikely, of an unusually precocious onset of PFIC2, treatment with dexamethasone was started, and the patient’s clinical improvement was surprising. However, contrary to our hypothesis, no mutations responsible for PFIC or for the majority of classical neonatal cholestatic diseases were detected. Surprisingly, a genetic analysis showed a homozygous mutation in the HFE gene, responsible for HH, a disease that usually induces iron overload during adulthood (10).

**FIGURE 2:**
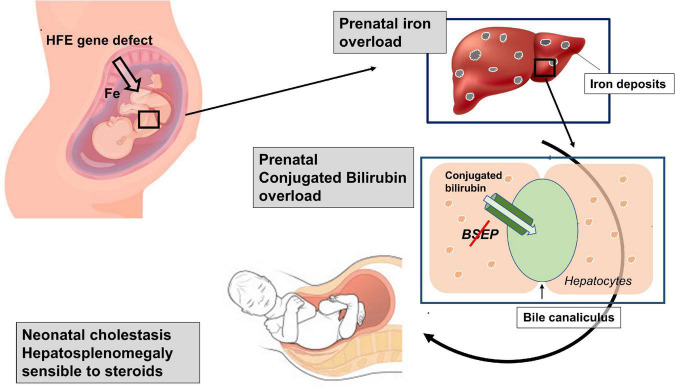
Schematic representation of the suggested double overloads: The genetic defect predisposes the fetus to an intrauterine iron overload, able to inhibit the function of the bile salt export pump (Bsep). The consequent bilirubin overload, which probably already occurred in the uterus, is probably responsible for the hepatosplenomegaly development.

In summary, our patient showed clinical signs consisting of an iron overload, compatible with an HFE defect, in the prenatal life, when a similar clinical picture is usually induced by an autoimmune disorder. The coexistence in the same patient of two different diseases, a first autoimmune (with an atypical clinical course) and a second genetic, both characterized by potential iron overload, appeared highly unlikely. Therefore, although it is difficult to exactly reconstruct the pathogenesis of such cholestasis, we can hypothesize a possible sequence of events. First, it is indisputable that our patient is affected by a genetic defect causing hepcidin deficiency, a key regulator of the iron transport across the placenta (11), that predisposes him to accumulate iron. Even though in adult patients, homozygous or heterozygous for the missense variant His63Asp, the risk of developing clinical iron overload is relatively low (12), the effect that this variant can have on placental function is, to our knowledge, unknown. Second, it is possible that the risk to develop an iron overload during the intrauterine life in this patient was favored by the maternal iron supplementation. The observation that HFE-knockout mice present iron accumulation in the liver more often than their wild-type counterparts if the maternal dietary iron intake is high (13) demonstrates that fetal genotype can affect prenatal iron acquisition. Accordingly, iron supplements should not be routinely given to pregnant women with HFE-related HH (14). We hypothesize that iron supplementation during the mother’s pregnancy favored a progressive iron overload in a fetus not sufficiently protected due to the hepcidin deficiency. Third, prenatal iron overload might also explain the occurrence of the consequent intrahepatic cholestasis and splenomegaly. In fact, in animal models, iron liver overload has been demonstrated to affect bile elimination through the downregulation of both BSEP and MRP2 apical transporters (15). Interestingly, when intrahepatic cholestasis develops, splenomegaly is a common finding (16). Therefore, the reconstruction of the patient’s clinical history suggests a combination of two overloads: first an intrauterine iron overload, capable of inhibiting the synthesis and function of enzymatic systems responsible for the elimination of conjugated bilirubin, and a subsequently conjugated bilirubin overload already occurring in the uterus, and probably responsible of hepatosplenomegaly development (Figure 2). For both such overloads, the newborn received a specific treatment. Iron overload was ameliorated through numerous blood phlebotomies performed for diagnostic tests, which were never accompanied by red blood cell transfusions or iron supplementation. Cholestatic overload (and consequent hepatosplenomegaly) was successfully and luckily treated with steroids thanks to the induction of the enzymatic system usually downregulated by iron accumulation. In addition, we cannot exclude the possibility that steroid efficacy might be related to its anti-inflammatory action, considering the liver inflammation induced by bile acid toxicity (17).

If our reconstruction of pathogenic events is correct, this newborn would be, to our knowledge, the first patient presenting HH with prenatal onset. The high incidence of the HFE genetic defect suggests that other infants with the same defect may have suffered from iron overload during intrauterine life but remained undiagnosed because HH is not usually included in panels for the differential diagnosis of neonatal cholestasis (2, 4). In our patient, the neurologic outcome was characterized by the persistence of axial hypotonia, EEG, and MR abnormalities. Considering that high concentrations of bile acids are toxic to various organs, including the central nervous system (18, 19), we cannot exclude that a more precocious diagnosis and treatment with steroids could have improved the neurological outcome.

## Conclusion

Although no firm conclusions can be drawn from a single clinical case, this experience suggests that HH could have played a role in the induction of this serious cholestasis already during the intrauterine life. Therefore, HH ought to be included in the panel of the possible causes of neonatal cholestasis, and steroids ought to be added to the pharmacological armamentarium for treating specific conditions which cause cholestasis in newborns.

## Data Availability Statement

The raw data supporting the conclusions of this article will be made available by the authors, without undue reservation.

## Ethics Statement

Ethical review and approval was not required for the study on human participants in accordance with the local legislation and institutional requirements. Written informed consent to participate in this study was provided by the participants’ legal guardian/next of kin. Written informed consent was obtained from the individual(s), and minor(s) legal guardian/next of kin, for the publication of any potentially identifiable images or data included in this article.

## Author Contributions

LF coordinated the clinical decisions on the patient, conceptualized and designed the study, drafted the initial manuscript, and critically reviewed it for important intellectual content. ST, FL, and RS collected the data, participated in the interpretation of data, and reviewed and revised the manuscript. AC and AM designed the data collection instruments, coordinated and supervised the data collection, performed genetic analysis, and reviewed and revised the manuscript. All authors approved the final manuscript as submitted and agreed to be accountable for all aspects of the work.

## Conflict of Interest

The authors declare that the research was conducted in the absence of any commercial or financial relationships that could be construed as a potential conflict of interest.

## Publisher’s Note

All claims expressed in this article are solely those of the authors and do not necessarily represent those of their affiliated organizations, or those of the publisher, the editors and the reviewers. Any product that may be evaluated in this article, or claim that may be made by its manufacturer, is not guaranteed or endorsed by the publisher.

## References

[1] Moyer V Freese DK Whitington PF Olson AD Brewer F Colletti RB Guideline for the evaluation of cholestatic jaundice in infants: recommendations of the North American Society for Pediatric Gastroenterology, Hepatology, and Nutrition. *J Pediatr Gastroenterol Nutr.* (2004) 39:115–28. 10.1097/00005176-200408000-00001 15269615

[2] Ranucci G Della Corte C Alberti D Bondioni MP Boroni G Calvo PL Diagnostic approach to neonatal and infantile cholestasis: a position paper by the SIGENP liver disease working group. *Dig Liver Dis.* (2022) 54:40–53. 10.1016/j.dld.2021.09.011 34688573

[3] Feldman AG Whitington PF. Neonatal hemochromatosis. *J Clin Exp Hepatol.* (2013) 3:313–20. 10.1016/j.jceh.2013.10.004 25755519 PMC3940210

[4] Chen HL Wu SH Hsu SH Liou BY Chen HL Chang MH. Jaundice revisited: recent advances in the diagnosis and treatment of inherited cholestatic liver diseases. *J Biomed Sci.* (2018) 25:75. 10.1186/s12929-018-0475-8 30367658 PMC6203212

[5] Linton KJ. Lipid flopping in the liver. *Biochem Soc Trans.* (2015) 43:1003–10. 10.1042/BST20150132 26517915

[6] Thompson R Strautnieks S. BSEP: function and role in progressive familial intrahepatic cholestasis. *Semin Liver Dis.* (2001) 21:545–50. 10.1055/s-2001-19038 11745042

[7] Warskulat U Kubitz R Wettstein M Stieger B Meier PJ Häussinger D. Regulation of bile salt export pump mRNA levels by dexamethasone and osmolarity in cultured rat hepatocytes. *Biol Chem.* (1999) 380:1273–9. 10.1515/BC.1999.162 10614819

[8] Fardel O Payen L Courtois A Vernhet L Lecureur V. Regulation of biliary drug efflux pump expression by hormones and xenobiotics. *Toxicology.* (2001) 167:37–46. 10.1016/s0300-483x(01)00456-5 11557128

[9] Engelmann G Wenning D Herebian D Sander O Droge C Kluge S Two case reports of successful treatment of cholestasis with steroids in patients with PFIC-2. *Pediatrics.* (2015) 135:e1326–32. 10.1542/peds.2014-2376 25847799

[10] Kowdley KV Brown KE Ahn J Sundaram V. ACG clinical guideline: hereditary hemochromatosis. *Am J Gastroenterol.* (2019) 114:1202–8. 10.14309/ajg.0000000000000315 31335359

[11] McDonald EA Gundogan F Olveda RM Bartnikas TB Kurtis JD Friedman JF. Iron transport across the human placenta is regulated by hepcidin. *Pediatr Res.* (2020): [Online ahead of print], 10.1038/s41390-020-01201-y 33069164 PMC8052381

[12] Gochee PA Powell LW Cullen DJ Du Sart D Rossi E Olynyk JK. A population-based study of the biochemical and clinical expression of the H63D hemochromatosis mutation. *Gastroenterology.* (2002) 122:646–51. 10.1016/s0016-5085(02)80116-0 11874997

[13] Balesaria S Hanif R Salama MF Raja K Bayele HK McArdle H Fetal iron levels are regulated by maternal and fetal Hfe genotype and dietary iron. *Haematologica.* (2012) 97:661–9. 10.3324/haematol.2011.055046 22180422 PMC3342966

[14] European Association For The Study Of The Liver. EASL clinical practice guidelines for HFE hemochromatosis. *J Hepatol.* (2010) 53:3–22. 10.1016/j.jhep.2010.03.001 20471131

[15] Prasnicka A Lastuvkova H Alaei Faradonbeh F Cermanova J Hroch M Mokry J Iron overload reduces synthesis and elimination of bile acids in rat liver. *Sci Rep.* (2019) 9:9780. 10.1038/s41598-019-46150-7 31278332 PMC6611795

[16] Baker A Kerkar N Todorova L Kamath BM Houwen RHJ. Systematic review of progressive familial intrahepatic cholestasis. *Clin Res Hepatol Gastroenterol.* (2019) 43:20–36. 10.1016/j.clinre.2018.07.010 30236549

[17] Woolbright BL Jaeschke H. Inflammation and cell death during cholestasis: the evolving role of bile acids. *Gene Expr.* (2019) 19:215–28. 10.3727/105221619X15614873062730 31253204 PMC6827039

[18] McMillin M DeMorrow S. Effects of bile acids on neurological function and disease. *FASEB J.* (2016) 30:3658–68. 10.1096/fj.201600275R 27468758 PMC5067249

[19] Grant SM DeMorrow S. Bile acid signaling in neurodegenerative and neurological disorders. *Int J Mol Sci.* (2020) 21:5982. 10.3390/ijms21175982 32825239 PMC7503576

